# A longitudinal rat forelimb model for assessing *in vivo* neuromuscular function following extremity reperfusion injury

**DOI:** 10.21203/rs.3.rs-5582098/v1

**Published:** 2025-01-29

**Authors:** Omar A. Selim, Aida K. Sarcon, Mehmet Furkan Tunaboylu, Chunfeng Zhao, Steven L. Moran

**Affiliations:** 1 Department of Orthopedic Surgery, Mayo Clinic, Rochester, MN.; 2 T32 Musculoskeletal Research Training Program, Mayo Clinic, Rochester, MN.; 3 Division of Plastic Surgery, Mayo Clinic, Rochester, MN

**Keywords:** Postreperfusion syndrome, forelimb ischemia, preclinical model, rhabdomyolysis, skeletal muscle reperfusion

## Abstract

Rhabdomyolysis following revascularization of the ischemic upper extremity can lead to life- & limb-threatening sequelae. In the context of replantations and vascularized composite allografting, a reconstructive procedure usually reserved for upper limb amputees, prolonged tissue ischemia is detrimental to extremity functional recovery. Currently, validated survival small animal models of extremity reperfusion injury that permit longitudinal assessment of limb function are lacking. To date, studies that evaluated reperfusion injury-induced neuromuscular impairment rely on terminal *ex vivo* procedures and do not provide clinically translatable measurements. Furthermore, it is unclear if upper extremity musculature exhibits a different ischemic threshold compared to the lower limb given the relatively rare incidence of upper limb ischemia. Here, we present a reliable rat model of extremity post-reperfusion syndrome (PRS) that comprehensively recapitulates the biochemical hallmarks of rhabdomyolysis secondary to upper extremity reperfusion injury and allows for monitoring *in vivo* upper limb function using clinically relevant electrodiagnostic and kinematic metrics. In addition to inducing severe metabolic derangements, our forelimb PRS provided insights on gross motor and electrophysiological alterations upper-extremity reperfusion injury. We identify gait coordination parameters such as stride frequency and forelimb-hindlimb coordination index and electrophysiological metrics including compound muscle action potential amplitude as objective, non-invasive outcome measures for limb function assessment in small animal models of extremity PRS. This comprehensive, validated functional model can serve as an invaluable tool to evaluate therapeutics or preconditioning regimens to attenuate PRS and mitigate resulting neuromuscular dysfunction.

## 1. INTRODUCTION:

Post-reperfusion syndrome (PRS) is a life-threatening clinical syndrome that can occur secondary to prolonged tourniquet use, acute limb ischemia, and transplantation of vascularized muscle flaps and allografts.^1–3^ In addition to inducing local limb tissue damage, severe extremity reperfusion injury can result in systemic inflammatory response, multiple systems organ failure, and even death.^4^ From a functional perspective, extremity IRI is a key prognostic factor in the field of upper extremity vascularized composite tissue allotransplantation (VCA) that may render limb allografts non-functional.^5^ Furthermore, the vigorous acute inflammatory response elicited by tourniquet-induced IRI have shown to contribute to quadriceps muscle weakness and impaired range of motion immediately following total knee arthroplasty (TKA) procedures.^6,7^

Currently, the *in vivo* evaluation of IRI-induced neuromuscular impairment in preclinical models remains an area of further investigation since most studies have largely focused on biochemical and histological evaluation, and survival small animal models integrating physiologic measures of muscle and nerve recovery are lacking.^8^ Previous studies have attempted *ex vivo* techniques such as single muscle fiber contractility (SFMC) testing and motor endplate potentials (EPPs) to evaluate ischemic muscle damage following reperfusion injury. ^9,10^However, these approaches are considered terminal requiring animal sacrifice for postmortem muscle tissue assessment and require long preparation time, highly specialized equipment, and expertise, which limits their bedside use. Therefore, we sought to validate a survival model that provides clinically relevant metrics of extremity function following reperfusion injury.

Structurally, the forelimb in animals, especially rodents, differs in neurovascular supply, musculoskeletal anatomy, and biomechanics than the hindlimb to allow for the execution of highly dexterous movements such as grasping, reaching, and object manipulation. These functional and structural differences have promoted several authors to explore the forelimb model for studying upper limb allotransplantation and other relevant neuromuscular procedures such as nerve repairs, etc. that would allow for more accurate representations of upper limb movements in humans.^11–13^ Furthermore, our interest in upper extremity composite allotransplantation and disease triggered us to establish a survival model that can closely mimic the postreperfusion syndrome (PRS) following revascularization of the ischemic upper limb of patients while permitting functional assessment.

In this work, we established a clinically representative postreperfusion syndrome (PRS) model following ischemic injury of the rat upper extremity and employed *in vivo* gait coordination metrics and electrophysiological to characterize neuromuscular dysfunction. Furthermore, we validated the extremity reperfusion injury model using non-contact laser perfusion imaging, clinical serum biomarkers, and cytokine multiplexing.

## 3. RESULTS

### 3.1. A digit exsanguinating tourniquet results in acute forelimb ischemia, grossly edematous changes, and polymorphonuclear infiltration in skeletal muscles upon extremity reperfusion.

The application of a digit exsanguinating tourniquet device at the rat shoulder joint resulted in acute forelimb ischemia denoted by severe forepaw pallor (Figure 2A) and coldness throughout the ischemia phase of the protocol. LSCI demonstrated a two-fold reduction in perfusion units (P.U.) following tourniquet application compared to steady-state measurements (356.30 ± 30.49 a.u. vs. 127.5 ± 12.37 a.u.; p = <0.0001) (Figure 2B & 2C). Moreover, forelimb blood flow could be re-established successfully upon tourniquet release indicating lack of permanent vascular injury or ‘no reflow’ phenomenon secondary to prolonged tourniquet use in this model (356.30 ± 30.49 a.u. vs. 390.00 40.93 a.u.; p = 0.7571) (Figure 2B).

Acute forelimb ischemia was further validated by evaluating brachial artery pulsatile waveforms using digital doppler assessment (Figure 2B; right column). Normally, the rat brachial arterial waveform is triphasic. Upon tourniquet placement at the shoulder joint, immediate disappearance of this typical waveform was observed validating both clinical and LSCI findings (Figure 2B). Furthermore, our I/R protocol resulted in massive forelimb swelling at both at 24- and 48-hours following extremity reperfusion compared to the contralateral normal limb (24-h I/R: 1.70 ± 0.07 vs. 1.00 ± 0.01, p = <0.0001; 48-h I/R: 1.69 ± 0.07 vs. 1.00 ± 0.01, p = <0.0001) (Figure 2D).

### 3.2. Forelimb post reperfusion syndrome leads to extensive muscle damage, renal injury, and electrolyte derangement:

Serum CK showed significant elevation only in rats subjected to 3 hours of reperfusion compared to WT group (15,735.00 ± 4,803.00 U/L vs. 107.40 ± 25.27 U/L, p = 0.0003). Mean CK was elevated in both 24 h I/R (1020.00 ± 525.30, p = 0.9867) and 48 h I/R (317.40 ± 56.73 U/L, p = 0.9998) groups compared to WT rats, however, not statistically significant (Figure 3C). Compared to WT animals, serum LDH was significantly elevated in both 3 h I/R (1919.00 ± 415.90 U/L vs. 158.30 ± 17.00 U/L, p = 0.0002) and 24 h I/R (1279.00 ± 304.10 U/L vs. 158.30 ± 17.00 U/L p = 0.015) groups. However, no statistical difference was found between WT rats and animals that underwent 48 I/R (707.9 ± 311.8 U/L vs. 158.30 ± 48.07 U/L, p = 0.3366) (Figure 3C). Serum AST and ALT levels were significantly elevated among all groups compared to WT rats (Figure 3C). Potassium (K^+^) was significantly elevated only in animals that underwent 3-hours reperfusion compared to WT group (5.21 ± 0.26 mEq/L vs. 4.05 ± 0.09 mEq/L, p = 0.0345) (Figure D, i).

In our model, we observed kidney dysfunction only in rats subjected to 3-h and 24-h reperfusion (Figure 3B). Creatinine levels were significantly higher in the 3 h I/R group only compared to WT rats (0.88 ± 0.17 mg/dL vs. 0.31 ± 0.01 mg/dL, p = 0.0016). Another renal function marker, blood urea nitrogen (BUN), was significantly higher both in 3 h and 24 h I/R groups (Figure 3B).

### 3.3. Postreperfusion syndrome secondary to forelimb ischemia triggers systemic inflammatory response syndrome (SIRS) and immune dysfunction.

Multiplexed cytokine profiling of the 3h I/R injury group revealed a pro-inflammatory response characterized by significantly elevated myelopoietic growth factors and granulocyte chemoattractants; G-CSF (*p*= 0.0242), GM-CSF (*p*=0.0029), GRO/KC/CINC-1 (*p*=0.0010), MIP-1α (*p*=0.0412), and Eotaxin (*p*=0.0113). Additionally, five proinflammatory cytokines were significantly elevated in the 3h I/R injury group: IL-1α (*p*=0.0149), IL-5 (*p*=0.0229), IL-6 (*p*=0.0014), TNF-α (*p*=0.0096), INF-γ (*p*=0.021) (Figure 4). Three regulatory or anti-inflammatory cytokines were also significantly elevated in animals that underwent 3 hours reperfusion injury compared to WT rats: IL-10 (p = 0.0154), IL-13 (p = 0.0024), and EGF (p = 0.0478).

In contrast, animals that underwent 24-hours reperfusion showed a dampened immune response with several cytokines significantly reduced compared to normal subjects (Figure 4). These included MIP-2 (p = 0.0141), IL-1β (p = 0.0330), IL-2 (p = 0.0219), IL-18 (p = 0.0294), VEGF, and eotaxin (p = 0.0471).

### 3.4. Forelimb reperfusion injury induces motor coordination deficits:

Complete forelimb paralysis and dragging gait was noted upon recovery of the animals in their reperfusion phase (Figure 5A). Longitudinal gait kinematics revealed altered paw area dynamics, gait symmetry and coordination (Figure 5B) compared to the contralateral uninjured forelimb. Furthermore, there was significantly reduced stride length, increased stride frequency, and increased FL-HL coordination reflecting asymmetrical gait behavior following reperfusion of the rat forelimb up to 2 weeks following injury.

### 3.5. Impairment of forelimb neuromuscular transmission:

A significant reduction of the CMAP negative peak amplitude was observed immediately upon forelimb reperfusion; over 85% reduction in peak amplitude (Figure 6B & 6C). Almost two thirds of animals had an evocable CMAP at 3 hours following reperfusion (4.37 ± 2.19 mV). However, one animal failed to recover CMAP in the 2-week follow-up period. At the end of the 2 weeks follow-up period, mean CMAP values recovered to approximately 25% of the preinjury measurements (Figure 6Ci). Additionally, CMAP amplitude at 2-weeks post-injury was significantly higher than at 24- and 48-hours following reperfusion injury indicating onset of neuromuscular recovery (2-weeks vs. 24 hours: 7.07 ± 1.42 mV vs. 2.95 ± 1.21 mV, p = 0.04; 2-weeks vs. 48 hours: 7.07 ± 1.42 mV vs. 3.48 ± 1.19 mV, p = 0.02). There were no dramatic changes in the onset latency and CMAP duration across all timepoints (Figure 6C).

Stimulated needle single fiber electromyography (SFEMG) of the extrinsic wrist flexors of the injured forelimb also revealed loss of single fiber action potentials (SFAPs) across the entire time course of the experiment (Figure 7B). Immunohistochemical analysis of the NMJ further confirmed motor endplate denervation characterized by loss of the presynaptic nerve endings and preservation of the postsynaptic acetylcholinesterase receptors (Figure 7D).

## 4. DISCUSSION:

In the present study, we employed clinically relevant neuromuscular and biochemical outcome measures to validate a functional rodent model of forelimb reperfusion injury. We used a facile technique to induce acute limb ischemia that achieved widespread systemic and local tissue damage without the use of specialized equipment such as hemorrhoidal ligator set or the need for surgical expertise to devascularize the limb as has been employed in prior hindlimb ischemia/reperfusion injury (IRI) studies.

Small animal models have been developed to study peripheral extremity reperfusion injury.^14–17^ However, these models do not provide clinically meaningful insights on functional impairment following induction of extremity reperfusion.^10^ Although addressing the detrimental systemic consequences is fundamental to IRI research, the ensuing neuromuscular dysfunction cannot be neglected. Furthermore, current models are exclusively based on inducing ischemia of hindlimb skeletal muscles and/or their innervating nerves such as the sciatic or tibial nerves. It is known that several factors exist that influence the ischemic tolerance of skeletal muscle tissue including muscle location, ischemic limb collateral blood flow, temperature, fiber type, and metabolic profile (glycolytic vs. oxidative), etc. and, hence, their susceptibility to reperfusion injury.^18,19^ Although upper limb ischemia is less common than lower extremity ischemia,^20^ the severity of rhabdomyolysis injury following reperfusion of the ischemic upper extremity remains undetermined both in clinical and preclinical literature.

Rhabdomyolysis following IRI is generally associated with significant elevations in creatine kinase (CK), lactate dehydrogenase (LDH), and aminotransferases.^22^ In line with other hindlimb I/R studies, our upper extremity reperfusion injury protocol was associated with a comparable altered serum enzymes profile albeit with some differences in the magnitude and peak times. In our study, rats of the 3-hours reperfusion group showed the most severe derangements in serum biochemical markers (Figure 3). Forelimb I/R injury was associated with CK values that were twofold greater than hindlimb I/R models with similar ischemia/reperfusion durations.^14,23^ However, our results were almost ten-fold greater than ligation-induced hindlimb I/R models.^24^ Although serum CK is highly specific to skeletal muscle tissue, the enzyme exists in other organ tissues such as cardiac muscle, brain, and kidneys. Furthermore, it has a very short half-life (2–4 hours), which makes monitoring muscle damage beyond the hyperacute phase difficult.^25^ This was evident in animals that underwent 24- and 48-hours of reperfusion that showed non-significant CK levels compared to wild-type controls, indicating rapid clearance of CK from the systemic circulation (Figure 3C).

LDH and the transaminases were elevated in almost all experimental groups compared to wild type controls. However, both ALT and AST were the last to normalize and remained significantly higher in the 48-hours reperfusion group indicating a longer t ½ compared to CK and LDH. Based on these results, we can infer the temporal changes in serum enzyme levels following forelimb reperfusion injury as depicted in the schematic (Figure 3E). Furthermore, the drastic differences in the serum levels of these enzymes reflect their relative abundance in skeletal muscle tissue and confirms the typical response to muscle disease. Although the levels of serum muscle enzymes highly correlate with the intensity of skeletal muscle membrane damage and necrosis, a consensus lacks on laboratory definitions for muscle injury based on serum enzymes results. Nevertheless, some references define CK values more than 5 times the upper normal limit or >1000–5000 U/L as a diagnostic criterion for rhabdomyolysis.^25,26^ Additionally, CK of 17,000 U/L is indicative of serious limb ischemia in humans and values >15,000 U/L are likely to be associated with renal failure.^27^

Hyperkalemia is a fatal sequel of rhabdomyolysis that can cause lethal cardiac arrhythmias. We observed clinically significant hyperkalemia (30% increase) only in animals that underwent 3-hours of forelimb reperfusion. Clemens et al. documented a 150% increase in potassium levels during the 2-hour reperfusion phase of a swine model of 2-hour bilateral hindlimb ischemia.^28^ Another study by Tricarico and coworkers reported over 90% increase in serum potassium following 3 hours of reperfusion in a 4-hour ligation-induced unilateral hindlimb ischemia rat model.^29^ However, some authors did not report significant changes in potassium levels following tourniquet-induced hindlimb reperfusion injury with comparable ischemia times as in our study.^14,23,30^ These results show that the severity of hyperkalemia appears to correlate proportionately with ischemia durations and bilateral vs. unilateral limb involvement, and can be affected by the I/R technique as well.

Another serious complication of extremity PRS is acute kidney injury (AKI). In the present study, our results demonstrate that renal impairment was evident in the early phases of forelimb reperfusion injury (3- and 24-hours). Similarly, renal injury appears to be the most severe around the 3-hours reperfusion timepoint as other studies have also demonstrated in hindlimb models of traumatic rhabdomyolysis.^14,23,31^

Furthermore, our forelimb PRS model resulted in approximately 85% increase in proximal forelimb girth in animals that underwent 24- and 48-hours of reperfusion. In comparison with lower extremity reperfusion injury studies, forelimb swelling induced using our I/R protocol is apparently more severe than previously published reports of hindlimb reperfusion injury. For instance, Cearra et al. and Deune et al. reported 30% and 38% increase in the hindlimb cross-sectional area after 24 h reperfusion, respectively. Another study also showed a 15% increase in the perimeter of the rat lower limb following tourniquet-ischemia.

Previous studies of the systemic cytokine response showed that serum IL-6, IL-8, TNF-α, and not IL-1β, were elevated following lower extremity ischemia/reperfusion in humans.^45^ Ferreira et al. also showed that patients with chronic limb-threatening ischemia had 11 higher serum cytokines: IL1ra, IL-6, IL-8, IL12 p70, G-CSF, IP-10, MCP-1, MIP-1α, PDGF-β, RANTES, and TNF-α compared to patients with claudication only.^34^ Additionally, Orfany and coworkers demonstrated that MCP-1, MCP-5 (monocyte chemoattractant protein 5), thymus and activation-regulated chemokine (TARC), and macrophage inflammatory protein 3a (MIP 3a) are significantly increased at 24 hours of hindlimb reperfusion in a mice model.^35^

Our serum multiplex cytokine assay showed elevated levels of 13 proinflammatory and regulatory cytokines only in animals that underwent 3 hours of reperfusion. These included IL-1α, IL-6, IL-10, IL-13, IL-18, TNF-α, MIP-1α, G-CSF, INF-γ, etc. Prior hindlimb I/R studies showed variable peaks for the cytokine response with some reporting systemic inflammation persisting up to 24 hours following extremity reperfusion injury. Interestingly, the cytokine profile of animals that underwent 24- and 48-hours of reperfusion reveal a suppressed immune response where multiple cytokines were significantly reduced or could not be detected compared to wild type rats (Figure 4). This phenomenon of dampened immune state has been described by some authors as sterile post-traumatic immunosuppression and can occur in multiple scenarios including following major surgical trauma, solid organ ischemia/reperfusion, polytrauma, etc..^36^ Although the mechanisms contributing to this condition remain not properly elucidated, a class of molecules known as damage-associated molecular patterns (DAMPs) has been shown to play a role in inducing systemic immunosuppression following sterile trauma including post reperfusion syndrome.^36,37^

From a clinical standpoint, experimental animal models of extremity PRS should translate pathological and biochemical damage into functional impairment or recovery. Most extremity I/R studies are limited by the lack of rigorous and clinically meaningful *in vivo* functional assessment. The digital exsanguinating tourniquet used in this study resulted in forelimb paralysis and a dragging (shuffling) gait. One report also observed hindlimb dragging in a mice model of 2-hour tourniquet I/R at 24 hours following reperfusion, however, gait coordination metrics were not reported.^35^ Dragging gait is typically associated with reduced stride length and slower gait speed in humans.^38^ The former finding was captured on gait kinematics in addition to increased stride frequency of the injured forelimb throughout the entire experiment duration with a trend towards recovery to baseline values by the 2^nd^ week.

Additionally, the analytical software of the DigiGait^™^ system computes a gait symmetry index. The gait symmetry is also referred to as the FL-HL coordination and it mathematically compares FL and HL step frequency.^39^ Normal gait, by definition, has a FL-HL coordination value of approximately 1.0 (Figure 5). However, this index was significantly increased following forelimb reperfusion in a similar manner to what has been reported for rodent models of spinal cord and arthritis injury.^39,40^ At the end of the 2-weeks follow-up period, partial recovery of shoulder motion could be visually observed (Supplementary video), and there was a trend towards normalization of the gait coordination metrics Although we attempted to use gripping force to measure hand function, this test was not successful due to the profound distal paralysis present.

The application of *in vivo* electrophysiology represents a sensitive tool to precisely assess neuromuscular dysfunction in limb ischemia-reperfusion injury. Previous work by Schoen et al. highlighted the inability to elicit compound muscle action potential (CMAP) in hindlimb extensor muscles that underwent ischemia of 3 hours using intramuscular needle electromyography.^41^ This can occur due to the relatively small uptake area of the needle electrode tip that samples very limited number of motor units and can miss viable muscle fibers. We also observed a similar phenomenon when we attempted to use single fiber needle electromyography (SFEMG) to interrogate the SFAP of extrinsic wrist flexors in this study. Although CMAP represents a summation of skeletal muscle electrical activity, SFAP can be helpful to determine perturbations in NMJ transmission *in vivo* due to the high selectivity of the single fiber electrode owing to its small 25-μm diameter recording surface.^42^ However, SFAPs with acceptable waveform criteria could not be provoked after the I/R procedure, possibly due to challenges with capturing viable motor units. Although some animals started to recover SFAP in some motor units by the 1^st^ and 2^nd^ week post-injury, over 90% of the synapses lacked an evocable motor unit potential despite examining at least 8 NMJs per setting.

For forelimb CMAP evaluation, we instead relied on non-invasive percutaneous surface electrodes that were able to detect subtle changes in the CMAP of extrinsic wrist flexor muscles and precisely monitor their progressive recovery over the course of injury (Figure 6). The CMAP size (area or amplitude) is indicative of the number and size of its constituent motor units (MUs). Diminution of the CMAP amplitude (or area) can result from loss of MUs as in axonal injury, reduced MU size (myofiber injury in myopathy), or an endplate disorder causing presynaptic NMJ transmission failure.^43^

Acute peripheral nerve compression and ischemia have been shown to cause dramatic reductions in the CMAP with varying recovery depending on the insult severity.^44,45^ Furthermore, Iida et al. reported over 95% reduction in CMAP amplitude (10.86 mV to 0.31 mV) in an isolated sciatic-tibial nerve 4-hours ligation-induced ischemia model.^46^ They also demonstrated further reductions in CMAP amplitude (0.08 mV) one week postoperatively highlighting additional damage secondary to reperfusion injury. The CMAP results of our tourniquet model also corroborates their findings as we similarly observed a precipitous drop in the CMAP amplitude during the 3-hours reperfusion period (27.83 mV to 4.37 mV). However, we only noted further reductions in the CMAP amplitude during the 24- and 48-hours following tourniquet release (2.95 mV & 3.48 mV, respectively) highlighting additional MU damage due to reperfusion. Lastly, the onset of CMAP amplitude recovery was noticeable by the 2^nd^ week post-injury (7.07 mV) similar to their findings, which occurred between 14–28 days.

We do acknowledge the limitations of our study. First, we relied mostly on systemic biochemical changes to validate reperfusion injury. This is due to challenges associated with analyzing multiple muscles of the forelimb, and studying one muscle could result in sampling bias and influence results adversely. More importantly, serum profiling is more readily accessible and clinically applicable than analyzing skeletal muscle tissue in the healthcare setting. In addition to its invasive nature, biopsying skeletal muscle tissue does not predict global disease activity of an otherwise systemic condition.

Second, our follow-up duration for functional evaluation is relatively short and did not capture the full recovery timeframe to baseline. Furthermore, determining the isolated effect of reperfusion injury on skeletal muscle function alone in a survival model is implausible as the entire motor unit is vulnerable to ischemic insult and denervation of the NMJ following tissue reperfusion.^47,48^ We further believe that tourniquet-induced compression neurapraxia accentuated the motor impairment observed in this model, which made functional assessment secondary to tissue ischemia only a technical challenge.

## 5. CONCLUSION

We validated and characterized a comprehensive, reliable upper extremity model of clinically relevant post-reperfusion syndrome (PRS) that revealed significant neuromuscular transmission failure (NTF) coupled with systemic sequelae of traumatic rhabdomyolysis. Gait coordination dynamics and non-invasive compound muscle action potential (CMAP) monitoring can be reliably used to assess neuromuscular recovery in extremity PRS models. Muscle injury, electrolyte abnormality, and renal impairment is most remarkable at 3- and 24-hours following reperfusion of the rat forelimb.

## 6. METHODS:

### 6.1. Animals and ethical approval:

**Figure 1:**
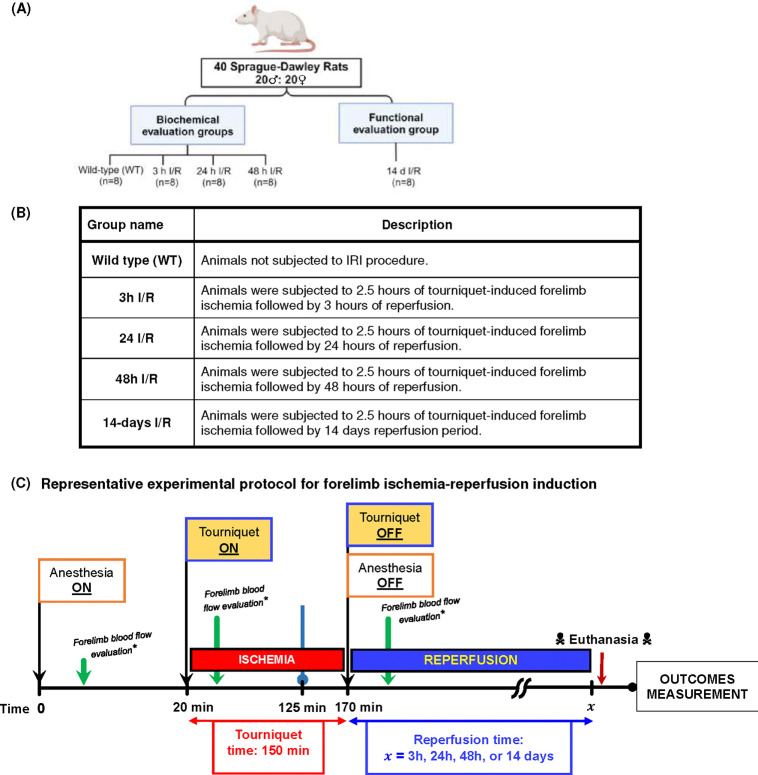
Study design and experimental protocol for rat forelimb PRS induction: A) & B) Study design and description of experimental groups. C) Representative experimental protocol for creating postreperfusion syndrome model secondary to forelimb ischemia-reperfusion.

All experimental procedures adhered to guidelines approved by the Mayo Clinic Institutional Animal Care and Use Committee (IACUC) (A00005926-21), and complied with the ARRIVE (Animal Research: Reporting of In Vivo Experiments) guidelines. Male and female Sprague-Dawley rats (Envigo, Indianapolis, IN, USA), aged one year and weighing between 300–500 grams, were selected for the study (Figure 1A & 1B). These rats were randomly assigned to either a functional evaluation group (n=8) or a biochemical assessment group (n=32). Rats were housed individually in a temperature-controlled environment, with a 12-hour light/dark cycle, and provided with standard rodent chow and water ad libitum.

### 6.2. Forelimb Post-Reperfusion Syndrome (PRS) Model

To induce the forelimb ischemia-reperfusion injury, rats were anesthetized with 3% isoflurane for induction and then maintained at 1.5% isoflurane via a small animal anesthesia machine (VetEquip, USA). The animal’s respiratory rate and body temperature were continuously monitored throughout the procedure. A thermal infrared heating pad (Kent Scientific, Torrington, CT, USA) was used to maintain the body temperature at 37°C, and fluids (10 mL of normal saline) were administered subcutaneously to prevent dehydration.

The left forelimb was chosen for the induction of ischemia, and the procedure was conducted in a sterile manner. Initially, the forelimb was exsanguinated by elevating it for 5 minutes, during which time the paw turned pale and cold, indicating a lack of blood flow. A self-exsanguinating silicone tourniquet (Tourni-Cot^®^, Mar-Med, MI, USA) was then applied just proximal to the shoulder joint, causing the cessation of blood flow to the forelimb.

The tourniquet was maintained for a period of 150 minutes. The choice of this duration was influenced by attempts to reduce anesthetic exposure time while achieving a clinically meaningful ischemic insult to the extremity tissue. A doppler ultrasonography probe (Dopplex^®^ DMX Digital Doppler, Huntleigh Healthcare, UK) was used to detect any peripheral pulses during ischemia and assess the restoration of blood flow during reperfusion. At the end of the ischemia phase, tourniquet was released restoring blood flow to the limb (Figure 1C).

### 6.3. Evaluation of forelimb perfusion

#### 6.3.1. Full-field laser speckle contrast imaging (LSCI):

A commercially available laser speckle contrast imaging platform (RFLSI-ZW, RWD Life Science, Guangdong, China) was utilized for quantifying cutaneous microcirculatory blood flow in our rodent model as an indirect measure of the upper extremity perfusion. The region-of-interest (ROI) analyzed for blood flow assessment was anatomically defined by the angiosome provided by the rat brachial artery. This method measures changes in laser speckle contrast and provides continuous blood flow data. The rat’s forelimb was imaged for a 10-minute interval post-reperfusion to track recovery.

The region of interest (ROI) was selected based on the major arterial distribution of the forelimb, including the brachial, ulnar, and median arteries, covering the region from the antecubital fossa to the fingertips. This ROI was chosen to represent both the proximal and distal areas of the limb, which could exhibit differing rates of reperfusion.

### 6.4. Gait kinematics

To assess the functional recovery of the forelimb after ischemia-reperfusion injury, gait kinematics were recorded using the DigitGait^™^ system (Mouse Specifics Inc., Boston, MA, USA). The system employs a motorized transparent treadmill belt over which the animal ambulated, while a video camera placed underneath captured the rat’s movement. The data were analyzed using the DigitGait^™^ proprietary software, which utilizes advanced algorithms to calculate key parameters such as stride length, stride frequency, and limb coordination. These gait parameters provide an indirect measure of the forelimb motor function. Pre-injury baseline gait data were collected for each rat, and subsequent recordings were made at specified time points following reperfusion (24 hours, 48 hours, and 1 week post-injury). This allowed for longitudinal tracking of motor function, with a focus on any deficits in forelimb coordination and muscle strength.

### 6.5. In vivo forelimb electrophysiology

#### 6.5.1. Nerve Conduction Studies (NCS)

Evoked compound muscle action potentials (CMAPs) of the antebrachium wrist flexors were recorded according to a previously reported method described by Harrigan et al. 2019 using a clinical electrodiagnostic system (Nicolet VikingQuest EMG/NC/EP (Natus^®^ Medical Incorporated, Middleton, WI).^49^ CMAP represents the summation of motor unit action potential (MUAPs) of the interrogated muscle motor units following peripheral nerve stimulation, and is not an action potential per se. Nevertheless, CMAP provides a non-invasive, clinically applicable, and quantitative measure of skeletal muscle function and innervation.

Briefly, the active recording (red; G1) and reference (G2, black) electrodes were placed over the wrist flexor muscles bulk immediately distal to the elbow joint and the wrist joint and metacarpus, respectively (Figure 6A). A disposable adhesive ground plate electrode (Natus^®^ Medical Incorporated, Middleton, WI) was wrapped around the tail. Disposable insulated 28G monopolar EMG needles (902-DMF37-TP, Natus^®^ Medical Incorporated, WI) were used as the stimulating anode and cathode electrodes. The anode electrode was subcutaneously placed rostral to the pectoralis muscle, and the cathode was caudally inserted over the mid pectoralis muscle with 1 cm distance between both electrodes (Figure 6A).

CMAP responses were recorded by stimulating the brachial plexus with increasing intensity from 1–20 mA with a duration of 0.1 ms until a maximum response was achieved. Maximal CMAP response was confirmed by the lack of increase in CMAP amplitude following application of supramaximal stimulation (120% of previous stimulation intensity or 44 mA current intensity). Frequency; 1Hz Filter settings 10Hz-10 kHz. Measurements were obtained at a constant screen sensitivity and duration setting (Sensitivity: 100 mV, 5 mV per division; Duration: 10 ms, 1 ms per division). The best of three reproducible waveforms was recorded for each nerve conduction study. The onset latency, negative peak amplitude, and total CMAP duration were recorded for each tracing and averaged to obtain a mean value for each animal and timepoint.

#### 6.5.2. Single Fiber Electromyography (SFEMG)

To assess the neuromuscular junction (NMJ) function in the forelimb following ischemia-reperfusion injury, Single Fiber Electromyography (SFEMG) was employed. The Nicolet VikingQuest system (Natus^®^ Medical Incorporated, Middleton, WI, USA) with SFEMG needles was used to record action potentials from individual muscle fibers. A frequency of 10 Hz was used for stimulation, and each muscle fiber action potential was assessed for the presence of jitter, which indicates synaptic transmission deficits.

### 6.6. Forelimb girth measurement and edema index:

**Figure 2:**
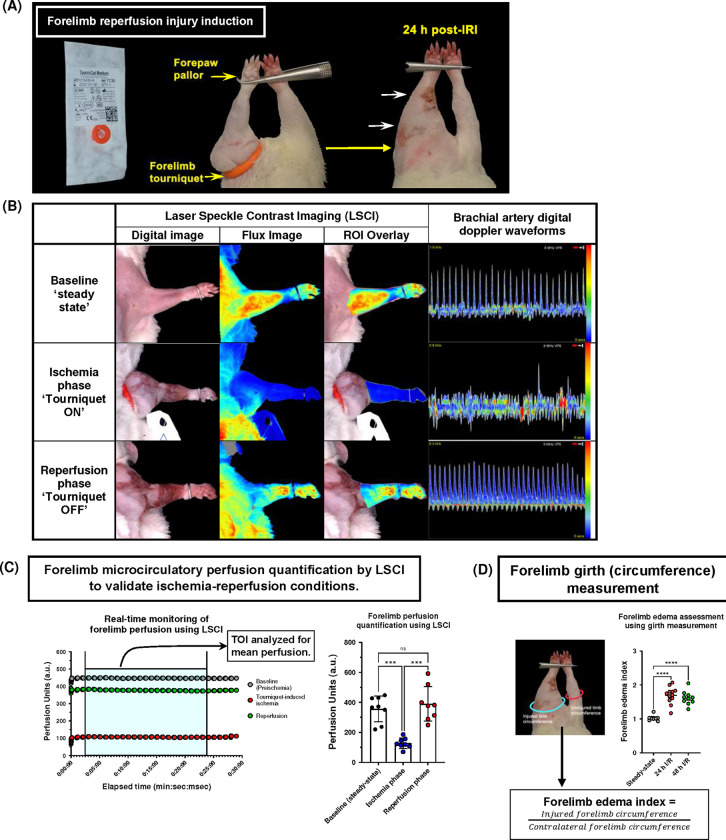
An exsanguinating digital tourniquet device effectively induces acute forelimb ischemia and clinically relevant post-reperfusion syndrome in a rat model: A) Placement of an exsanguinating digit tourniquet (Torni-cot^™^) at the shoulder joint level results in acute forelimb ischemia in a rat model. Massive forelimb edema at 24 hours following tourniquet release secondary to post-reperfusion syndrome. B) Digital images of perfusion assessment using laser speckle contrast imaging and digital doppler waveform analysis under different ischemia-reperfusion conditions. A region of interest (ROI) is selected distal to the tourniquet placement site to evaluate tissue perfusion and confirm effective induction of ischemia. D) Non-invasive forelimb perfusion quantification using laser speckle contrast imaging (LSCI). Representative curve data of real-time monitoring of forelimb microcirculatory perfusion, and a time of interest (TOI) devoid of motion artefacts is selected for mean perfusion units’ analysis (left). Quantification and statistical analysis of arbitrary units (a.u.) of forelimb perfusion during baseline (steady state), tourniquet-induced ischemia, and reperfusion conditions; n=8; data presented as mean ± SEM. Statistical comparison was done using one-way ANOVA with Tukey’s post-hoc analysis. E) Evaluation of forelimb edema using forelimb girth measurement and forelimb edema index in rats subjected to 24h and 48 I/R injury (n=10–12). Data shown as mean ± SEM.***P<0.001; ****P< 0.0001; ns: non-significant.

**Figure 3:**
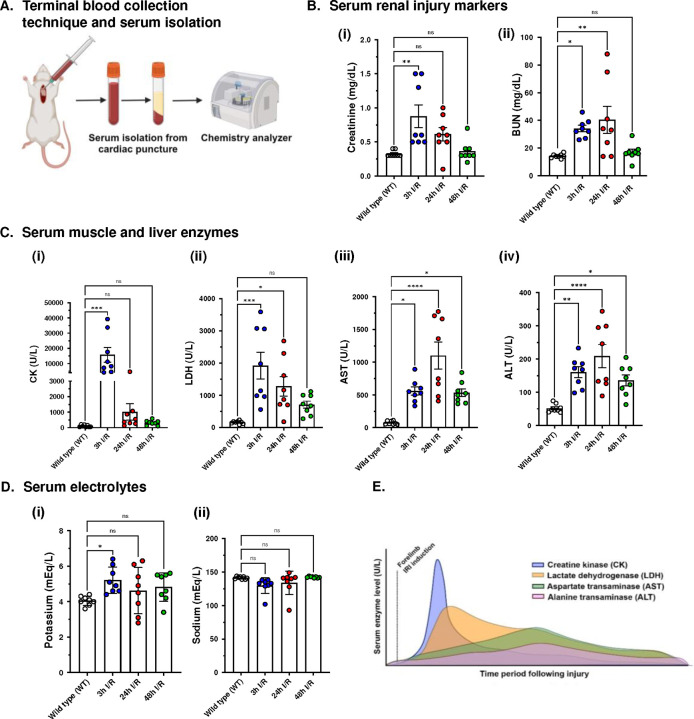
Forelimb post-reperfusion syndrome results in extensive biochemical & metabolic derangements in a rat model: (A) Terminal blood collection via cardiac puncture for serum biomarkers analysis of skeletal muscle and renal damage. Serum chemistry profile of four groups (n=32; n=8 each) (B) Renal function markers: (i) serum creatinine; (ii) serum blood urea nitrogen (BUN). (C) Muscle and liver enzymes: (i) serum creatine kinase (CK); serum lactate dehydrogenase (LDH); (iii) serum aspartate transaminase (AST); (iv) serum alanine transaminase (ALT). (D) Electrolytes: (i) serum potassium; (ii) serum sodium. (E) Schematic delineating putative temporal changes in serum muscle enzymes following forelimb ischemia-reperfusion injury. (F) Skeletal muscle and renal histology. P values were determined by one-way ANOVA followed by Dunnett’s post-hoc test. Data presented as mean ± standard error of mean (SEM). *P<0.05; **P<0.01; ***P<0.001; ****P<0.0001: ns = non-significant.

**Figure 4:**
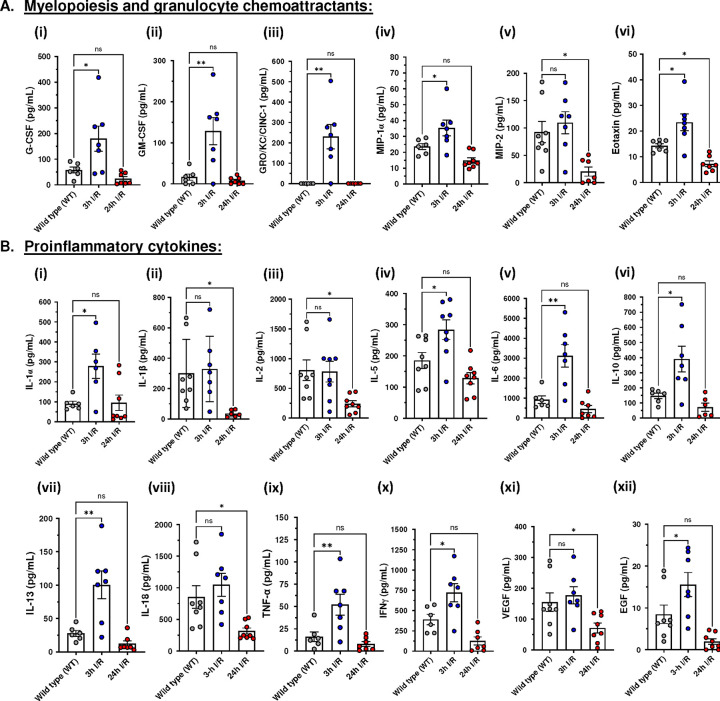
Forelimb ischemia-reperfusion injury triggers systemic inflammatory response syndrome (SIRS) and immune dysfunction: Luminex^®^ cytokine 27-analytes profiling of serum extracted from wild-type (WT) rats (n=6–8), rats subjected to 3 hours of forelimb reperfusion injury (3h I/R, n=6–7), and rats subjected to 24 hours of reperfusion injury (24h I/R, n=6–8). (A) Myelopoietic and granulocyte chemokines: (i) granulocyte-colony stimulating factor (G-CSF); (ii) granulocyte monocyte-colony stimulating factor (GM-CSF); (iii) chemokine ligand 1 (CXCL1)/also known as GRO/KC/CINC-1; (iv) macrophage inflammatory protein-1 alpha (MIP-1α); (v) macrophage inflammatory protein-2 (MIP-2); (vi) eotaxin. (B) Proinflammatory cytokines: (i) interleukin-1 alpha (IL-1α); (ii) interleukin-1 beta (IL-1β); (iii) interleukin-2; (iv) interleukin-5 (IL-5); (v) interleukin-6 (IL-6); (vi) interleukin-13 (IL-13); (viii) interleukin-18 (IL-18); (ix) tumor necrosis factor-alpha (TNF-α); (x) interferon-gamma (IFN-γ); (xi) vascular endothelial growth factor (VEGF); (xii) epidermal growth factor (EGF). Statistical comparisons between groups with one-way ANOVA with Dunnett’s correction post hoc analysis. Multiplex cytokine assay of serum following reperfusion injury. P values were determined by one-way ANOVA followed by Dunnett’s post-hoc test. Data presented as mean ± standard error of mean (SEM). *P<0.05; **P<0.01; ns = non-significant.

**Figure 5:**
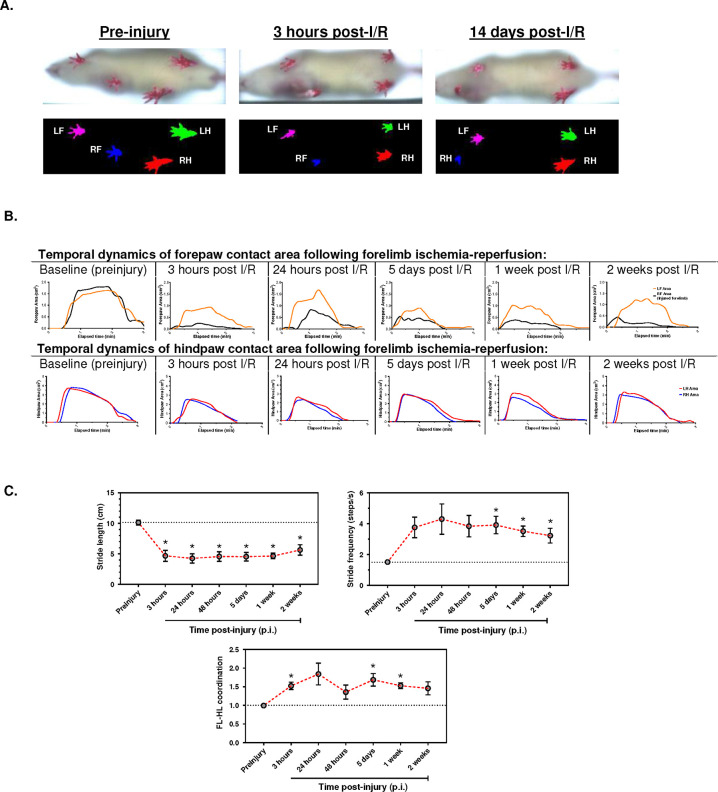
Motor coordination is impaired following forelimb reperfusion injury: A) Top: representative ventral views of a test rat at different timepoints post-injury depicting gross alterations in paw positioning after ischemia-reperfusion. Bottom: Software-digitized paw prints generated by the DigiGait high-speed ventral plane videography system. B) Representative dynamic gait signals of a rat at a treadmill speed of 15 cm/s; c) Gait coordination parameters (n=5–8): top: Gait symmetry (also known as Forelimb [FL]-Hindlimb [HL] coordination index); middle: stride frequency; bottom: stride length. Repeated measure one way ANOVA (mixed-effects model). Data presented as mean ± standard error of mean (SEM). * P<0.05. FL-HL: forelimb/hindlimb. RF: right forelimb. LF. Left forelimb. RH: right forelimb. LH: left hindlimb.

**Figure 6:**
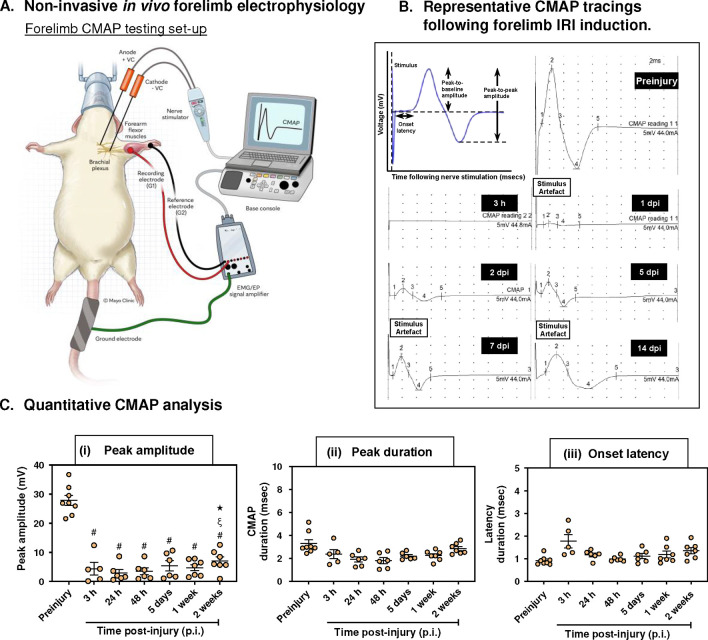
Forelimb IRI model induces persistent conduction block: A) Experimental setup for nerve conduction and compound muscle action potential testing of the rat forelimb using Nicolet VikingQuest EMG/NCS/EP system. B) Representative CMAP tracings of the rat wrist extrinsic flexors before IRI induction and at different timepoints immediately following forelimb reperfusion injury. C) Quantitative analysis of CMAP waveform amplitude, area, and duration. Data presented as mean ± SEM (n=5–8). Repeated measure one-way ANOVA (mixed-effects model) was used for statistical analysis. #: statistically significant compared to baseline (pre-injury) values. ξ: statistically significant compared to 24 h values post-injury. ✶: statistically significant compared to 48 h post-injury. P<0.05 was considered statistically significant.

**Figure 7:**
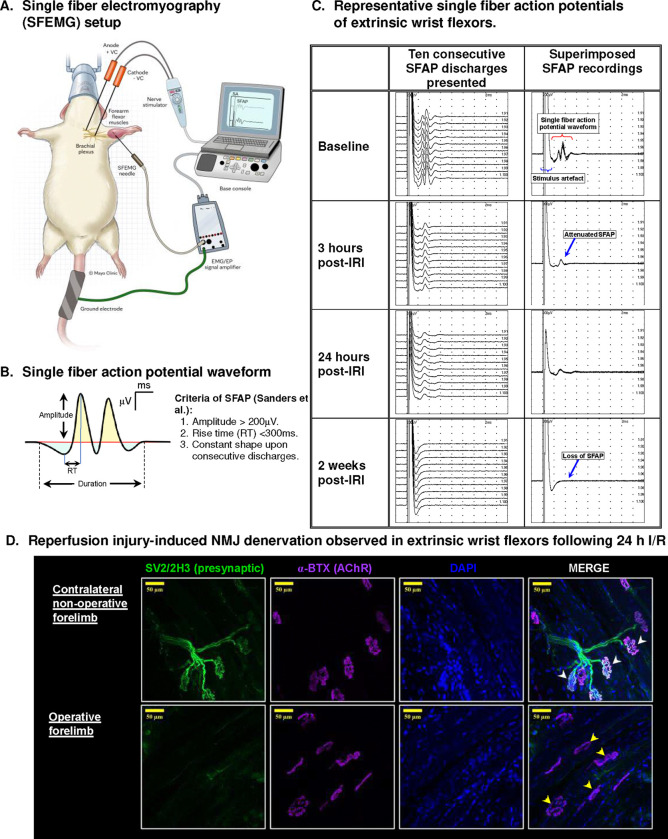
Loss of motor unit action potentials and NMJ denervation following forelimb PRS: A) Stimulated single fiber electromyography (SFEMG) setup for evaluating forelimb single fiber action potential: B) Single fiber action potential waveform: shown is a waveform of action potentials recorded from two muscle fibers. A typical motor unit potential is characterized by an initial positive deflection (shaded in cyan) followed by a steep negative deflection (shaded in light yellow). C) Representative SFAP tracings at different timepoints: baseline SFAP from extrinsic wrist flexors before tourniquet application (top); subsequent loss of motor unit action potentials following reperfusion injury of the forelimb. D) Immunohistochemical analysis of the neuromuscular junction (NMJ) of the flexor digitorum profundus (FDP), major extrinsic wrist flexor muscle, showing NMJ denervation with loss of the presynaptic structures including nerve ending and presynaptic vesicles at 24 hours following reperfusion. White arrow shows normal NMJ structures of the contralateral normal muscle; Yellow arrow shows denervated NMJs with preservation of the postsynaptic receptors.

To validate interstitial edema following forelimb IRI, the circumference of the forelimb distal to the tourniquet placement site was measured. Under anesthesia, animals were placed in a supine position with the injured forelimb outstretched and pinned in position. The forelimb circumference of both injured and uninjured limbs was measured three times using a suture thread at the level of the olecranon process (elbow joint). A micrometer or a ruler was used to measure the length of each thread, and the average was recorded. Forelimb edema index was calculated by dividing the mean injured forelimb girth by the contralateral uninjured forelimb circumference (Figure 2D).

### 6.7. Tissue and blood sampling:

At the end of the experiment, animals were euthanized humanely via exsanguination. Terminal blood collection was obtained by a cardiac puncture for serum analysis. Whole blood was allowed to clot at room temperature for at least 30 minutes before serum was isolated by centrifugation at 4° C for 15 minutes at 1800g. The serum samples were aliquoted in 200 𝜇L and stored in sterile plastic tubes at −80°C until use for serum clinical chemistry and cytokine profiling.

### 6.8. Serum chemistry:

To evaluate for the extent of muscle and renal injury, serum muscle enzymes including creatine kinase (CK), lactate dehydrogenase (LDH), aspartate transaminase (AST), and alanine transaminase (ALT) and the renal function markers, creatinine and blood urea nitrogen (BUN), and electrolytes, were quantified using an automated chemistry analyzer (Beckman Coulter, IDEXX Bioanalytics, Grafton, MA)

### 6.9. Serum cytokines profiling:

A multiplex rat cytokine/chemokine 27-plex discovery assay with Luminex^®^ xMAP technology was used to evaluate 27 markers in the serum according to manufacturer’s protocol (Eve Technologies, AB, Canada). The assay (Eotaxin | EGF | Fractalkine | IFNγ | IL-1α | IL-1β | IL-2 | IL-4 | IL-5 | IL-6 | IL-10 | IL-12p70 | IL-13 | IL-17A | IL-18 | IP-10 | GRO/KC | TNFα | G-CSF | GM-CSF | MCP-1 | Leptin | LIX | MIP-1α | MIP-2 | RANTES | VEGF-A) was performed in duplicates on undiluted serum samples.

### 6.10. NMJ immunohistochemistry and motor endplate imaging:

NMJ was immunohistochemically stained by fixing fresh flexor digitorum profundus skeletal muscle whole mounts in 4% paraformaldehyde (Electron Microscopy Services, Hatfield, PA) for 1 hour at room temperature. Muscles were then permeabilized using 2% Triton X-100 and blocked in 4% BSA/1% Triton-X before incubation with primary antibodies against SV2 (synaptic vesicle glycoprotein) and 2H3 (neurofilament) (Developmental Studies Hybridoma Bank, DSHB) at 4° C for 72 hours. Next, muscles were washed several times with 1X TBS before incubation with Alexa Fluor-555 conjugated bungarotoxin (Invitrogen, Carlsbad, CA) and anti-mouse Alexa Fluor 488 (Invitrogen, Carlsbad, CA). NMJs micrographs were then obtained using Carl Zeiss LSM 980 (Germany). Z-stacks were obtained and projected to maximum intensity using ImageJ (Fiji plugin).

### 6.11. Statistical Analysis

Data were analyzed using GraphPad Prism v10.2.3 (GraphPad Software, San Diego, CA, USA). Outliers were identified using the ROUT method, and one-way ANOVA followed by post-hoc testing (Dunnett’s or Tukey’s test) was used to compare between groups at different time points. Differences were considered statistically significant at a p-value of less than 0.05.

## Figures and Tables

**Table 1:** Representative DigitGait^™^ Metrics used in this study:

Gait index	Description

1. Paw area (cm^2^)	The maximal paw area in contact with the treadmill belt during the stance part of the step cycle.
2. Stride frequency	The mean number of times a paw touches the belt per second.
3. Stride length (cm)	The distance between initial contacts of the same paw in one complete stride.
4. FL-HL coordination or “gait symmetry index”	Software-generated index. It represents the sum of right and left FL stride frequency divided by the sum of left and right HL stride frequency. deviations from complete coordination, which is mathematically defined as 1.0

FL = forelimb; HL = hindlimb
